# Cysteines as Redox Molecular Switches and Targets of Disease

**DOI:** 10.3389/fnmol.2017.00167

**Published:** 2017-06-06

**Authors:** Annamaria Fra, Edgar D. Yoboue, Roberto Sitia

**Affiliations:** ^1^Department of Molecular and Translational Medicine, University of BresciaBrescia, Italy; ^2^Division of Genetics and Cell Biology, Vita-Salute San Raffaele UniversityMilan, Italy; ^3^Division of Genetics and Cell Biology, IRCCS San Raffaele Scientific InstituteMilan, Italy

**Keywords:** cellular redoxstasis, cysteine mutation, signaling pathways, disulfide bonding, protein misfolding

## Abstract

Thiol groups can undergo numerous modifications, making cysteine a unique molecular switch. Cysteine plays structural and regulatory roles as part of proteins or glutathione, contributing to maintain redox homeostasis and regulate signaling within and amongst cells. Not surprisingly therefore, cysteines are associated with many hereditary and acquired diseases. Mutations in the primary protein sequence (gain or loss of a cysteine) are most frequent in membrane and secretory proteins, correlating with the key roles of disulfide bonds. On the contrary, in the cytosol and nucleus, aberrant post-translational oxidative modifications of thiol groups, reflecting redox changes in the surrounding environment, are a more frequent cause of dysregulation of protein function. This essay highlights the regulatory functions performed by protein cysteine residues and provides a framework for understanding how mutation and/or (in)activation of this key amino acid can cause disease.

## Introduction

Amino acids are much more than mere building blocks of proteins: their different chemical properties dictate the catalytic activity of enzymes, protein half-life and a plethora of different post-translational modifications that govern protein function. This essay concentrates on the role of cysteine, a thiol containing amino acid that can participate in a variety of chemical reactions such as post-translational oxidative modifications. Many of them are reversible at physiological conditions, thereby allowing cysteine to act as a powerful molecular switch, akin to protein phosphorylation-dephosphorylation cycles. Hence, cysteine modifications are not limited to the well-known structural role of disulfide bonds in proteins synthesized in the endoplasmic reticulum (ER), but participate in fundamental intra- and inter-cellular signaling pathways. The downside of the pleiotropic reactivity of cysteines resides is their high susceptibility to undesired activation/inactivation in conditions of redox disequilibrium (either oxidative or reductive stress). In many genetic diseases and cancer, mutations can either directly target a cysteine or affect residues that contribute to maintain optimal cysteine pKa, accessibility and/or reactivity.

Here we briefly discuss redoxstasis in cell compartments and provide examples of disease—associated modifications/mutations of key cysteine residues.

## Cysteines Reactivity and Redox Homeostasis

**Figure 1:**
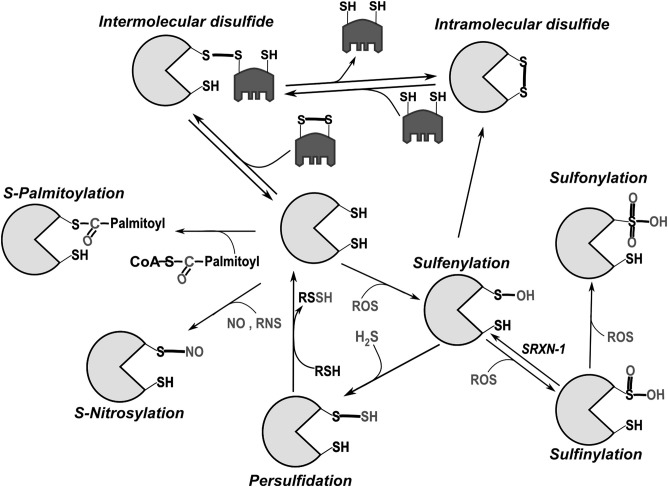
Main post-translational modifications of cysteines. Intermolecular disulfide bonds can be formed with another protein or low molecular weight thiols (like glutathione). In general, intramolecular bonds are inserted into a reduced protein by disulfide exchange with oxidized glutathione (GSSG) or another oxidized protein (e.g., Protein disulfide isomerase, PDI), through the formation of mixed disulfides. Oxidation by reactive oxygen species (ROS) initially leads to sulfenylation (SOH). Because of its relative instability, sulfenylated cysteine can promote intramolecular disulfide bond formation or additionally react with ROS leading first to sulfinylation (SO_2_H) and then to sulfonylation (SO_3_H). While SO_2_H can be reversed through the catalytic activity of the cytoplasmic enzyme sulfiredoxin-1 (SRXN-1; Biteau et al., 2003), SO_3_H is so far considered irreversible. Palmitoylation can also take place through creation of thioester bonds between palmitate and cysteine (Fukata and Fukata, 2010).

Redox reactions involve the gain (reduction) or loss (oxidation) of electrons in the reacting compounds. From its reduced form (SH), the sulfur atom of a cysteine residue can undergo a wide-range of oxidative modifications (Figure 1). Reactivity is greatly enhanced for cysteines whose thiol side chain is in the thiolate form, i.e., deprotonated at physiological pH (S^−^), and is influenced by structural factors (Ferrer-Sueta et al., 2011). Disulfide bonds stabilize the tertiary and/or quaternary structures of many proteins. They also serve as regulatory functional switches, a prototype being the activation of the bacterial transcription factor OxyR in response to oxidative stress (Zheng et al., 1998; Jo et al., 2015). Progressive cysteine oxidation by H_2_O_2_ leads to cysteine sulfenylation (SOH), sulfinylation (SO_2_H) and sulfonylation (SO_3_H). Among these, oxidation to SO_3_H is regarded as irreversible. S-sulfydration (also called persulfidation) can occur after reactions between derivatives of hydrogen sulfide (H_2_S) and thiols (Mishanina et al., 2015). Reactive nitrogen species (RNS) like nitric oxide (NO) react with some cysteines causing S-nitrosylation/nitrosation (Evangelista et al., 2013). Cysteines can also undergo lipid modifications including palmitoylation and prenylation or bind metals such as Zn, Fe and Cu. This latter property is crucial for formation of zinc fingers and iron-sulfur clusters (Oteiza, 2012; Rouault, 2015). Owing to their nucleophilic properties, thiolate groups also participate in non-redox reactions as in the catalytic groups of cysteine-proteases and ubiquitin ligases. For a detailed discussion on cysteine reactivity, its chemotypes and the methods for their detection, we refer to excellent reviews (Nagy, 2013; Paulsen and Carroll, 2013; Go et al., 2015).

**Figure 2:**
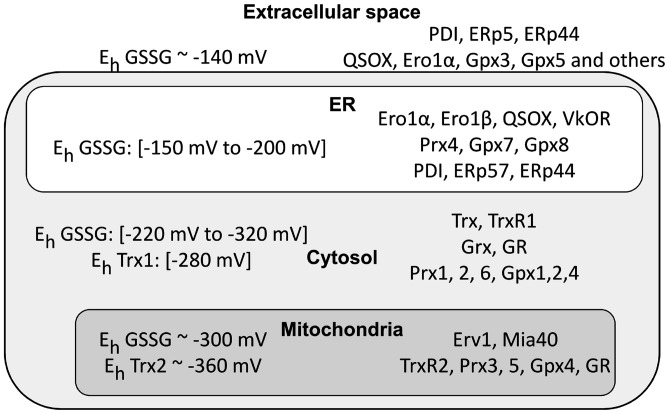
Cellular compartments differ in their redox poise. The left part of the figure summarizes the data available in the literature concerning glutathione redox potential values (E_h_ GSSG) in intra- and extra-cellular compartments. Depending on the cell types, physiological conditions and methods used, the results can vary rather significantly. There remains no doubt, however, that mitochondria and cytosol are far more reducing than the endoplasmic reticulum (ER) and extracellular space. Noteworthy, Thioredoxin 1 (Trx1) and Trx2 display a more reducing Kox, confirming their pivotal role in maintaining a suitable redox in the cytosol. Owing to the permeability of nuclear pores, the nucleus is likely to have values similar to the cytosol (values are from Gutscher et al., 2008; Jones and Go, 2010; Kojer et al., 2012; Birk et al., 2013; Kirstein et al., 2015). The right part highlights instead the main redox control systems in the cytosol, mitochondria and ER. Note the presence in the ER of proteins promoting formation of disulfide bonds (endoplasmic reticulum oxidoreductin 1 (Ero1), quiescin sulfhydryl oxidase (QSOX), PDI, ERp44, etc.) and also the absence of Glutathione reductase (GR), thus contributing to higher GSSG/GSH ratios in the ER compared to the cytosol.

Cysteine thiols are key players in conditions of oxidative stress. Most non-protein antioxidants as well as antioxidant enzymes are thiol based. Glutathione (GSH, γ-L-Glutamyl-L-cysteinylglycine) acts as a redox buffer and a cofactor of many enzymes including glutathione peroxidases (Gpx) that scavenge peroxides generating oxidized glutathione (GSSG). In humans, there are eight Gpxs, localized in different compartments (Brigelius-Flohé and Maiorino, 2013; Figure 2). Other key peroxide scavengers are peroxiredoxins (Prx; Perkins et al., 2015). Of the six human Prxs, two are localized in mitochondria and one in the ER. Thioredoxins (Trx) and glutaredoxins (Grx) reduce oxidized protein thiols. Oxidized Trx and Grx are reduced by Trx reductases (TrxR) and GSH, respectively (Holmgren, 1979; Mustacich and Powis, 2000; Fernandes and Holmgren, 2004). Glutathione reductase (GR) is also a key player for redox homeostasis, replenishing the GSH pool at the expense of GSSG. It is important to stress that both GR and TrxR rely on the NADPH/NADP system for their activity, thus establishing a link between the nicotinamide and thiol redox systems (Jones and Sies, 2015). Another important player is sulfiredoxin-1, an ATP-dependent enzyme capable of reducing sulfinylated proteins (Biteau et al., 2003; Mishra et al., 2015).

## Redox Compartmentalization

The organelles of eukaryotic cells can differ dramatically with respect to the redox poise of their various redox couples (Figure 2). The chemistry of primordial cells evolved in an oxygen-free atmosphere, and cytosolic and mitochondrial cysteines tend to remain in the reduced states (Go et al., 2015). Incidentally, this is why most cell-free protocols that recapitulate nuclear or cytoplasmic reactions include the addition of DTT or other reductants to work efficiently. *In vivo*, the establishment of stable disulfide bonds in these compartments is extremely unfavorable because of the combined reducing power of GSH, Grxs and Trxs.

However, there are places where disulfide bonds need to be inserted into selected proteins. For example, the Mia40/Erv1 relay allows formation of disulfide bonds in proteins and their import into the inter-membrane mitochondrial space, where the low Grx pool kinetically favor this process (Mesecke et al., 2005; Kojer et al., 2015; Erdogan and Riemer, 2017).

The redox couples present in organelles of the exocytic pathway display redox poises similar to the extracellular space. In the ER, nascent membrane and secretory proteins form disulfide bonds, preparing for their adult life in the oxidizing extracellular environments. Thus, oxidative power is needed in the ER, but redox conditions must be tightly controlled to allow isomerization or reduction of non-native disulfides. The occurrence of opposite reactions is guaranteed by protein relays capable of selectively inserting or removing disulfides (Fassio and Sitia, 2002; Hagiwara and Nagata, 2012). Oxidative folding is catalyzed by enzymes of the protein disulfide isomerase (PDI) superfamily, which receive oxidative power from oxidases like endoplasmic reticulum oxidoreductin 1 (Ero1) and quiescin sulfhydryl oxidase (QSOX; Bulleid and Ellgaard, 2011; Hudson et al., 2015). The human genome encodes for numerous PDI-like enzymes whose activity largely depends on the number of Trx-like domains and the redox potential of their CXXC motifs (Hatahet and Ruddock, 2009; Okumura et al., 2015). Depending on the surrounding redox and ionic conditions, oxidoreductases may oxidize, isomerize or reduce disulfides. In this wide range of activities, ERdJ5 is most suited for reducing disulfides (Dong et al., 2008; Ushioda et al., 2008). TrxR and import of cytosolic GSH have been proposed as reducing powers to prevent ER hyperoxidation (Molteni et al., 2004; Appenzeller-Herzog, 2011; Poet et al., 2017).

Disulfide interactions with PDI-like enzymes provide key quality control of the secretome, preventing the release of immature proteins. For instance, ERp44, captures proteins with exposed thiols and redox-active enzymes lacking suitable ER localization signals (e.g., Ero1, Prx4, Sumf1), retrieving them to the ER (Vavassori et al., 2013; Anelli et al., 2015).

## Cysteines as Redox Molecular Switches

Mechanisms ensuring tight redoxstasis control are present in the three cellular compartments where protein folding takes place (cytosol, ER and mitochondria) and they are intimately linked to protein quality control (Anelli et al., 2015). However, cells additionally exploit cysteine reactivity for purposes other than oxidative protein folding, namely as switches regulating signaling and adaptive responses.

A prototypic example is provided by the Nuclear factor erythroid 2-related factor 2 (Nrf2), a transcription factor whose nuclear translocation is prevented by interactions with Kelch-like ECH-associating protein 1 (Keap1). Upon oxidation, Keap1 dissociates from Nrf2, which can reach the nucleus and promote transcription of antioxidant response genes (Dinkova-Kostova et al., 2002). Recently, unexpected links between Nrf2, redox and ER stress emerged. Ire1 is a transmembrane protein that initiates the unfolded protein response upon accumulation of misfolded proteins in the ER lumen. Upon oxidative stress, Ire1 is sulfenylated and activates the Nrf2 pathway, abandoning its canonical ER stress sensing function (Hourihan et al., 2016). Thus, a subtle cysteine modification can shift the pathway to which a signal transducer is affiliated.

Redox modifications also play key roles in regulating protein tyrosine phosphorylation. Cysteine oxidative modifications such as SOH, disulfide formation and S-nitrosylation inhibit phosphatase and tensin homolog (PTEN) and other protein tyrosine phosphatases by interfering for example with their cysteine-dependent catalytic activity (Numajiri et al., 2011; Corcoran and Cotter, 2013; Pulido, 2015). As an example of physiological importance, abolishing this rheostat circuit dampens B lymphocyte activation and antibody production (Bertolotti et al., 2016). Moreover, a growing body of evidence supports the redox regulation of several tyrosine kinases, as described for c-Src (Giannoni and Chiarugi, 2014) and Janus kinase 2 (JAK2; Smith et al., 2012).

High mobility group protein B1 (HMGB1) is a DNA-binding nuclear protein that can be released by stressed cells. In the extracellular space, HMGB1 mediates inflammation or tissue repair, according to its redox state. If fully reduced, it binds to Advanced glycation end product-specific receptor (RAGE) and C-X-C chemokine receptor type 4 (CXCR4) and activates cell migration and autophagy. Upon formation of an intramolecular disulfide bond, HMGB1 binds Toll-like receptor 4 (TLR4)/MD-2 receptors complex and stimulates cytokine secretion. Sulphonylation then inactivates HMGB1, highlighting how a protein switches function depending on its cysteine redox state (Fiuza et al., 2003; Venereau et al., 2012; Vénéreau et al., 2015). Oxidation of a conserved cysteine residue also modulate the permeability of aquaporin 8 by reversibly inhibiting the transport of H_2_O_2_ and H_2_O across the membrane of stressed cells (Medraño-Fernandez et al., 2016).

Another example of redox-based functional re-targeting aimed to prevent protein aggregation is the induction of holdase activity in the ER chaperone Immunoglobulin heavy chain-binding protein (BiP) by cysteine oxidation (Wei et al., 2012; Wang et al., 2014). Similarly, Prx sulphinylation promotes formation of homo-oligomers endowed with chaperone activity (Jang et al., 2004; Hanzén et al., 2016).

Thus, cysteine modifications are key in many intra- and inter-cellular signaling and adaptive pathways. The sub-compartmental organization of redoxstasis, based on spatially constrained protein relays (Woo et al., 2010), and the low diffusibility of small redox active compounds such as H_2_O_2_ (Bienert and Chaumont, 2014) can explain how redox-dependent signals can propagate in the presence of powerful antioxidant systems.

## Cysteines and Diseases

### Secretory Proteins

Owing to the importance of structural and regulatory disulfide bonds in membrane and secretory proteins, mutations in luminal cysteines generally have dramatic consequences. Hence, acquisition or loss of a cysteine often causes retention of the mutated protein in the ER by thiol-mediated mechanisms (Anelli et al., 2015), with consequent loss or gain of function. The difficulty in forming the proper array of disulfide bonds in the cysteine-rich domains of many membrane receptors can lead to ER retention and degradation (loss of function), but also gain of function by interchain disulfide bonding that chronically activates signal transduction. An astonishing example comes from type 2A multiple endocrine neoplasia (MEN2A). This severe condition is often due to mutations in the cysteine-rich luminal portion of a tyrosine kinase receptor, RET, with strong genotype-phenotype correlations. The oncogenic hit is the formation of ligand-independent, covalent homodimers that constitutively deliver growth signals (Asai et al., 1995; Mulligan, 2014). RET malfunction can also lead to congenital abnormalities characterized by failure of neuroblast migration and defective maturation of the enteric nervous system (Hirschsprung disease), a condition that in some families coexisted with MEN2A (Takahashi et al., 1999; Frank-Raue et al., 2011).

Many genetic diseases are caused by gain or loss of a cysteine in secretory or membrane proteins. Aberrant thiol-mediated interactions via unpaired cysteines can directly provoke ER retention and aggregation besides misfolding. In Pelizaeus-Merzbacher disease, a myelination defect, a subgroup of mutations affecting the extracellular loop of the PLP/DM20 protein impair formation of intramolecular disulfide bridges and cause abnormal protein cross-links, ER retention and oligodendrocyte death (Dhaunchak et al., 2011). Similar mechanisms have been demonstrated in some forms of autism (Comoletti et al., 2004), color blindness (Patel et al., 2005) and von Willebrand disease (Wang et al., 2012).

Mutations of uromodulin, causing medullary cystic kidney disease/familial juvenile hyperuricemic nephropathy most often affect one of the 48 conserved cysteine residues (Rampoldi et al., 2003; Scolari et al., 2015). Cysteine mutations and aberrant disulfide bonding underlie the pathogenesis of CD40 deficiency (Lanzi et al., 2010), TNFR1-associated periodic fever syndrome (Lobito et al., 2006) and MiDY insulin-deficient diabetes (Liu et al., 2010). Mutations causing conformational alterations of alpha-1-antitrypsin make its only cysteine more prone to form aberrant disulfide bonds in the ER, thus facilitating the intracellular retention and polymerization of alpha-1-antitrypsin in Alpha-1-antitrypsin deficiency (AATD; Ronzoni et al., 2016).

Marinesco-Sjogren is a syndrome causing ataxia, intellectual disability and muscle weakness. This rare disease is caused by mutations in Sil1, a cofactor of BiP (Anttonen et al., 2005; Krieger et al., 2013). In yeast recovering from stress, Sil1 reduces oxidized Kar2, the paralog of human BiP, restoring its normal foldase activity (Siegenthaler et al., 2017). It remains to be seen whether and how mutations in Marinesco-Sjogren patients also impact the reductase function of Sil1.

### Cytosolic and Nuclear Proteins

In cytosolic proteins, cysteines can be direct targets of mutations, but more frequently they are dysregulated or inactivated by oxidative stress or other environmental conditions. Both mechanisms have been shown in Parkinson’s disease (PD). Parkin (PARK2) is an E3 ubiquitin ligase whose dysfunction causes accumulation of protein aggregates, endangering dopaminergic neurons (Charan and LaVoie, 2015). Parkin is highly expressed in the brain and frequently mutated in autosomal recessive juvenile PD (Biskup et al., 2008). These mutations often affect cysteines, causing loss of function and decreased stability of the enzyme (Wang et al., 2005; Seirafi et al., 2015). Parkin can also be inactivated by S-nitrosylation or sulphonylation (Chung et al., 2004; Meng et al., 2011). Recent studies describe interesting interplays between parkin oxidative modifications, its role in mitochondrial quality control and PD onset (Zhang et al., 2016). In dopaminergic neurodegenerative disorders, a key pathogenetic event is also the inactivation of tyrosine hydroxylase, a rate-limiting enzyme in dopamine and norepinephrine biosynthesis, by oxidative injury (Di Giovanni et al., 2012).

Mutations of the antioxidant superoxide dismutase gene (SOD1) are linked to about one fifth of the cases of familial amyotrophic lateral sclerosis, a degenerative disorder of motor neurons. Wild type SOD1 is a covalent disulfide-linked homodimer localized in part in the mitochondrial intermembrane space. Pathogenic SOD1 mutants form high molecular weight oligomers, inducing mitochondrial dysfunctions (Ferri et al., 2006; Magrané et al., 2009). Noteworthy, intermolecular disulfide cross-links and glutathionylation enhance mutant SOD1 aggregation (Cozzolino et al., 2008; Redler et al., 2011; McAlary et al., 2013), cysteine 111 being a key residue (Valle and Carrì, 2017).

In addition, Alzheimer’s disease (AD) is associated with thiol modifications, in particular S-nitrosylation. NO is produced in the brain by neuronal NO synthase (nNOS) and serves as a key second messenger for instance, regulating neuronal plasticity and survival (Nakamura and Lipton, 2016; Chong et al., 2017). However, aberrant S-nitrosylation of proteins such as PDI and glyceraldehyde-3-phosphate dehydrogenase (GAPDH) can occur in AD (Uehara et al., 2006; Zhao et al., 2015). S-nitrosylation of GAPDH enhances its binding to the ubiquitin ligase Siah1. GAPDH/Siah1 complexes accumulate in the nucleus triggering neuronal apoptosis via excessive protein degradation and trans-nitrosylation signaling cascades (Hara et al., 2005; Sen and Snyder, 2011; Nakamura and Lipton, 2013).

Numerous examples of “gain of cysteine” mutations are found in cancer, p53, KRAS and other oncogenes being preferred targets. The acquired cysteines cause decreased stability or impaired DNA binding of the tumor suppressor p53, while KRAS oncogenes are constitutively activated. Noteworthy, such acquired cysteines are potential targets for antitumor treatments (Visscher et al., 2016).

## Concluding Remarks

The multiple chemical reactions of cysteines and their reversibility in physiological conditions make them ideal tuneable devices for regulating protein function. Indeed, evolution has increasingly exploited the regulatory potential of cysteine chemistry as atmospheric oxygen became more abundant and complex multicellular organisms evolved. The frequency and conservation of this amino acid is indeed higher in mammals (>2% of the proteome) than in prokaryotes (0.5%). The examples provided in this essay reveal the pathophysiological relevance of cysteine redox modifications in the different compartments of human cells. Disulfide bonds prevail in the exocytic and endocytic compartments, organelles which are in direct contact with the oxidizing extracellular environment. These covalent bonds increase protein stability, facilitate quality control (Medraño-Fernandez et al., 2014) and underlie the functional regulation of many secreted proteins. A wider range of modifications acts in the cytosol and mitochondria, whose chemistry reflects their origin in an oxygen free atmosphere. Cysteine residues in these compartments are largely found in the reduced thiol/thiolate state, which permits regulation of protein function and activity by way of a wide-range of oxidative post-translational modifications. The redox gradients that form within and amongst cells hence provide ample opportunities to regulate signaling, transcription and other key biological processes. The price we pay is the many diseases caused by cysteine mutations or oxidative deregulation. Novel reagents (Chen et al., 2013; Kim et al., 2015; Bilan and Belousov, 2016; Wagener et al., 2016) are being developed to better understand cysteine modifications and their links with disease, ultimately offering ample practical exploitations (Nakamura and Lipton, 2016; Wani and Murray, 2017).

## Author Contributions

AF, EDY and RS discussed the concepts and pitfalls, and wrote the manuscript.

## Conflict of Interest Statement

The authors declare that the research was conducted in the absence of any commercial or financial relationships that could be construed as a potential conflict of interest.

## Abbreviations

AATD: Alpha-1-antitrypsin deficiency
BiP: Immunoglobulin heavy chain-binding protein
CFTR: Cystic fibrosis transmembrane conductance regulator
CXCR4: C-X-C chemokine receptor type 4
ER: Endoplasmic reticulum
ERAD: ER associated degradation
Ero1: Endoplasmic reticulum oxidoreductin 1
Gpx: Glutathione peroxidase
GR: Glutathione reductase
Grx: Glutaredoxin
GSH/GSSG: Reduced/oxidized glutathione
HMGB1: High mobility group protein B1
KEAP1: Kelch-like ECH-associated protein 1
MD2: Lymphocyte antigen 96
Nrf2: Nuclear factor erythroid 2-related factor 2
PDI: Protein disulfide isomerase
Prx: Peroxiredoxin
PTEN: Phosphatase and tensin homolog
QSOX: Quiescin sulfhydryl oxidase
RAGE/ARGE: Advanced glycosylation end product-specific receptor
ROS: Reactive oxygen species
SOD1: Superoxide Dismutase 1
TLR4: Toll-like receptor 4
TNF: Tumor necrosis factor
TNFR: TNF receptors
Trx: Thioredoxin
TrxR: Thioredoxin reductase
VkOR: Vitamin K epoxide reductase.

## References

[B1] Anelli T. Sannino S. Sitia R. (2015). Proteostasis and “redoxtasis” in the secretory pathway: tales of tails from ERp44 and immunoglobulins. Free Radic. Biol. Med. 83, 323–330. 10.1016/j.freeradbiomed.2015.02.020 25744412

[B2] Anttonen A.-K. Mahjneh I. Hämäläinen R. H. Lagier-Tourenne C. Kopra O. Waris L.. (2005). The gene disrupted in Marinesco-Sjögren syndrome encodes SIL1, an HSPA5 cochaperone. Nat. Genet. 37, 1309–1311. 10.1038/ng1677 16282978

[B3] Appenzeller-Herzog C. (2011). Glutathione- and non-glutathione-based oxidant control in the endoplasmic reticulum. J. Cell Sci. 124, 847–855. 10.1242/jcs.080895 21378306

[B4] Asai N. Iwashita T. Matsuyama M. Takahashi M. (1995). Mechanism of activation of the ret proto-oncogene by multiple endocrine neoplasia 2A mutations. Mol. Cell. Biol. 15, 1613–1619. 10.1128/MCB.15.3.1613 7532281 PMC230385

[B5] Bertolotti M. Farinelli G. Galli M. Aiuti A. Sitia R. (2016). AQP8 transports NOX2-generated H2O2 across the plasma membrane to promote signaling in B cells. J. Leukoc. Biol. 100, 1071–1079. 10.1189/jlb.2ab0116-045r 27256569

[B6] Bienert G. P. Chaumont F. (2014). Aquaporin-facilitated transmembrane diffusion of hydrogen peroxide. Biochim. Biophys. Acta 1840, 1596–1604. 10.1016/j.bbagen.2013.09.017 24060746

[B7] Bilan D. S. Belousov V. V. (2016). New tools for redox biology: from imaging to manipulation. Free Radic. Biol. Med. [Epub ahead of print]. 10.1016/j.freeradbiomed.2016.12.004 27939954

[B8] Birk J. Meyer M. Aller I. Hansen H. G. Odermatt A. Dick T. P.. (2013). Endoplasmic reticulum: reduced and oxidized glutathione revisited. J. Cell Sci. 126, 1604–1617. 10.1242/jcs.117218 23424194

[B9] Biskup S. Gerlach M. Kupsch A. Reichmann H. Riederer P. Vieregge P.. (2008). Genes associated with Parkinson syndrome. J. Neurol. 255, 8–17. 10.1007/s00415-008-5005-2 18787878

[B10] Biteau B. Labarre J. Toledano M. B. (2003). ATP-dependent reduction of cysteine-sulphinic acid by *S. cerevisiae* sulphiredoxin. Nature 425, 980–984. 10.1038/nature02075 14586471

[B11] Brigelius-Flohé R. Maiorino M. (2013). Glutathione peroxidases. Biochim. Biophys. Acta 1830, 3289–3303. 10.1016/j.bbagen.2012.11.020 23201771

[B12] Bulleid N. J. Ellgaard L. (2011). Multiple ways to make disulfides. Trends Biochem. Sci. 36, 485–492. 10.1016/j.tibs.2011.05.004 21778060

[B13] Charan R. A. LaVoie M. J. (2015). Pathologic and therapeutic implications for the cell biology of parkin. Mol. Cell. Neurosci. 66, 62–71. 10.1016/j.mcn.2015.02.008 25697646 PMC4492119

[B14] Chen Y.-J. Ching W.-C. Lin Y.-P. Chen Y.-J. (2013). Methods for detection and characterization of protein *S*-nitrosylation. Methods 62, 138–150. 10.1016/j.ymeth.2013.04.016 23628946

[B15] Chong C.-M. Ai N. Ke M. Tan Y. Huang Z. Li Y.. (2017). Roles of nitric oxide synthase isoforms in neurogenesis. Mol. Neurobiol. [Epub ahead of print]. 10.1007/s12035-017-0513-7 28421538

[B16] Chung K. K. K. Thomas B. Li X. Pletnikova O. Troncoso J. C. Marsh L.. (2004). S-Nitrosylation of Parkin regulates ubiquitination and compromises Parkin’s protective function. Science 304, 1328–1331. 10.1126/science.1093891 15105460

[B17] Comoletti D. De Jaco A. Jennings L. L. Flynn R. E. Gaietta G. Tsigelny I.. (2004). The Arg451Cys-neuroligin-3 mutation associated with autism reveals a defect in protein processing. J. Neurosci. 24, 4889–4893. 10.1523/jneurosci.0468-04.2004 15152050 PMC6729460

[B18] Corcoran A. Cotter T. G. (2013). Redox regulation of protein kinases. FEBS J. 280, 1944–1965. 10.1111/febs.12224 23461806

[B19] Cozzolino M. Amori I. Pesaresi M. G. Ferri A. Nencini M. Carrì M. T. (2008). Cysteine 111 affects aggregation and cytotoxicity of mutant Cu,Zn-superoxide dismutase associated with familial amyotrophic lateral sclerosis. J. Biol. Chem. 283, 866–874. 10.1074/jbc.m705657200 18006498 PMC2842925

[B20] Dhaunchak A. S. Colman D. R. Nave K.-A. (2011). Misalignment of PLP/DM20 transmembrane domains determines protein misfolding in Pelizaeus–Merzbacher disease. J. Neurosci. 31, 14961–14971. 10.1523/JNEUROSCI.2097-11.2011 22016529 PMC6623585

[B21] Di Giovanni G. Pessia M. Di Maio R. (2012). Redox sensitivity of tyrosine hydroxylase activity and expression in dopaminergic dysfunction. CNS Neurol. Disord. Drug Targets 11, 419–429. 10.2174/187152712800792938 22483306

[B22] Dinkova-Kostova A. T. Holtzclaw W. D. Cole R. N. Itoh K. Wakabayashi N. Katoh Y.. (2002). Direct evidence that sulfhydryl groups of Keap1 are the sensors regulating induction of phase 2 enzymes that protect against carcinogens and oxidants. Proc. Natl. Acad. Sci. U S A 99, 11908–11913. 10.1073/pnas.172398899 12193649 PMC129367

[B23] Dong M. Bridges J. P. Apsley K. Xu Y. Weaver T. E. (2008). ERdj4 and ERdj5 are required for endoplasmic reticulum-associated protein degradation of misfolded surfactant protein C. Mol. Biol. Cell 19, 2620–2630. 10.1091/mbc.E07-07-0674 18400946 PMC2397301

[B24] Erdogan A. J. Riemer J. (2017). Mitochondrial disulfide relay and its substrates: mechanisms in health and disease. Cell Tissue Res. 367, 59–72. 10.1007/s00441-016-2481-z 27543052

[B25] Evangelista A. M. Kohr M. J. Murphy E. (2013). S-Nitrosylation: specificity, occupancy and interaction with other post-translational modifications. Antioxid. Redox Signal. 19, 1209–1219. 10.1089/ars.2012.5056 23157187 PMC3785808

[B26] Fassio A. Sitia R. (2002). Formation, isomerisation and reduction of disulphide bonds during protein quality control in the endoplasmic reticulum. Histochem. Cell Biol. 117, 151–157. 10.1007/s00418-001-0364-0 11935291

[B27] Fernandes A. P. Holmgren A. (2004). Glutaredoxins: glutathione-dependent redox enzymes with functions far beyond a simple thioredoxin backup system. Antioxid. Redox Signal. 6, 63–74. 10.1089/152308604771978354 14713336

[B28] Ferrer-Sueta G. Manta B. Botti H. Radi R. Trujillo M. Denicola A. (2011). Factors affecting protein thiol reactivity and specificity in peroxide reduction. Chem. Res. Toxicol. 24, 434–450. 10.1021/tx100413v 21391663

[B29] Ferri A. Cozzolino M. Crosio C. Nencini M. Casciati A. Gralla E. B.. (2006). Familial ALS-superoxide dismutases associate with mitochondria and shift their redox potentials. Proc. Natl. Acad. Sci. U S A 103, 13860–13865. 10.1073/pnas.0605814103 16945901 PMC1557633

[B30] Fiuza C. Bustin M. Talwar S. Tropea M. Gerstenberger E. Shelhamer J. H.. (2003). Inflammation-promoting activity of HMGB1 on human microvascular endothelial cells. Blood 101, 2652–2660. 10.1182/blood-2002-05-1300 12456506

[B31] Frank-Raue K. Rybicki L. A. Erlic Z. Schweizer H. Winter A. Milos I.. (2011). Risk profiles and penetrance estimations in multiple endocrine neoplasia type 2A caused by germline RET mutations located in exon 10. Hum. Mutat. 32, 51–58. 10.1002/humu.21385 20979234

[B32] Fukata Y. Fukata M. (2010). Protein palmitoylation in neuronal development and synaptic plasticity. Nat. Rev. Neurosci. 11, 161–175. 10.1038/nrn2788 20168314

[B33] Giannoni E. Chiarugi P. (2014). Redox circuitries driving Src regulation. Antioxid. Redox Signal. 20, 2011–2025. 10.1089/ars.2013.5525 23901911

[B34] Go Y.-M. Chandler J. D. Jones D. P. (2015). The cysteine proteome. Free Radic. Biol. Med. 84, 227–245. 10.1016/j.freeradbiomed.2015.03.022 25843657 PMC4457640

[B35] Gutscher M. Pauleau A.-L. Marty L. Brach T. Wabnitz G. H. Samstag Y.. (2008). Real-time imaging of the intracellular glutathione redox potential. Nat. Methods 5, 553–559. 10.1038/nmeth.1212 18469822

[B36] Hagiwara M. Nagata K. (2012). Redox-dependent protein quality control in the endoplasmic reticulum: folding to degradation. Antioxid. Redox Signal. 16, 1119–1128. 10.1089/ars.2011.4495 22229892

[B37] Hanzén S. Vielfort K. Yang J. Roger F. Andersson V. Zamarbide-Forés S.. (2016). Lifespan control by redox-dependent recruitment of chaperones to misfolded proteins. Cell 166, 140–151. 10.1016/j.cell.2016.05.006 27264606

[B38] Hara M. R. Agrawal N. Kim S. F. Cascio M. B. Fujimuro M. Ozeki Y.. (2005). S-nitrosylated GAPDH initiates apoptotic cell death by nuclear translocation following Siah1 binding. Nat. Cell Biol. 7, 665–674. 10.1038/ncb1268 15951807

[B39] Hatahet F. Ruddock L. W. (2009). Protein disulfide isomerase: a critical evaluation of its function in disulfide bond formation. Antioxid. Redox Signal. 11, 2807–2850. 10.1089/ARS.2009.2466 19476414

[B40] Holmgren A. (1979). Glutathione-dependent synthesis of deoxyribonucleotides. Characterization of the enzymatic mechanism of Escherichia coli glutaredoxin. J. Biol. Chem. 254, 3672–3678. 34620

[B41] Hourihan J. M. Moronetti Mazzeo L. E. Fernández-Cárdenas L. P. Blackwell T. K. (2016). Cysteine sulfenylation directs IRE-1 to activate the SKN-1/Nrf2 antioxidant response. Mol. Cell 63, 553–566. 10.1016/j.molcel.2016.07.019 27540856 PMC4996358

[B42] Hudson D. A. Gannon S. A. Thorpe C. (2015). Oxidative protein folding: from thiol-disulfide exchange reactions to the redox poise of the endoplasmic reticulum. Free Radic. Biol. Med. 80, 171–182. 10.1016/j.freeradbiomed.2014.07.037 25091901 PMC4312752

[B43] Jang H. H. Lee K. O. Chi Y. H. Jung B. G. Park S. K. Park J. H.. (2004). Two enzymes in one; two yeast peroxiredoxins display oxidative stress-dependent switching from a peroxidase to a molecular chaperone function. Cell 117, 625–635. 10.1016/j.cell.2004.05.002 15163410

[B44] Jo I. Chung I.-Y. Bae H.-W. Kim J.-S. Song S. Cho Y.-H.. (2015). Structural details of the OxyR peroxide-sensing mechanism. Proc. Natl. Acad. Sci. U S A 112, 6443–6448. 10.1073/pnas.1424495112 25931525 PMC4443364

[B45] Jones D. P. Go Y.-M. (2010). Redox compartmentalization and cellular stress. Diabetes Obes. Metab. 12, 116–125. 10.1111/j.1463-1326.2010.01266.x 21029308 PMC3052693

[B46] Jones D. P. Sies H. (2015). The redox code. Antioxid. Redox Signal. 23, 734–746. 10.1089/ars.2015.6247 25891126 PMC4580308

[B47] Kim H.-J. Ha S. Lee H. Y. Lee K.-J. (2015). ROSics: chemistry and proteomics of cysteine modifications in redox biology. Mass Spectrom. Rev. 34, 184–208. 10.1002/mas.21430 24916017 PMC4340047

[B48] Kirstein J. Morito D. Kakihana T. Sugihara M. Minnen A. Hipp M. S.. (2015). Proteotoxic stress and ageing triggers the loss of redox homeostasis across cellular compartments. EMBO J. 34, 2334–2349. 10.15252/embj.201591711 26228940 PMC4570520

[B49] Kojer K. Bien M. Gangel H. Morgan B. Dick T. P. Riemer J. (2012). Glutathione redox potential in the mitochondrial intermembrane space is linked to the cytosol and impacts the Mia40 redox state. EMBO J. 31, 3169–3182. 10.1038/emboj.2012.165 22705944 PMC3400016

[B50] Kojer K. Peleh V. Calabrese G. Herrmann J. M. Riemer J. (2015). Kinetic control by limiting glutaredoxin amounts enables thiol oxidation in the reducing mitochondrial intermembrane space. Mol. Biol. Cell 26, 195–204. 10.1091/mbc.E14-10-1422 25392302 PMC4294668

[B51] Krieger M. Roos A. Stendel C. Claeys K. G. Sonmez F. M. Baudis M.. (2013). SIL1 mutations and clinical spectrum in patients with Marinesco-Sjogren syndrome. Brain 136, 3634–3644. 10.1093/brain/awt283 24176978

[B52] Lanzi G. Ferrari S. Vihinen M. Caraffi S. Kutukculer N. Schiaffonati L.. (2010). Different molecular behavior of CD40 mutants causing hyper-IgM syndrome. Blood 116, 5867–5874. 10.1182/blood-2010-03-274241 20702779

[B53] Liu M. Haataja L. Wright J. Wickramasinghe N. P. Hua Q.-X. Phillips N. F.. (2010). Mutant INS-gene induced diabetes of youth: proinsulin cysteine residues impose dominant-negative inhibition on wild-type proinsulin transport. PLoS One 5:e13333. 10.1371/journal.pone.0013333 20948967 PMC2952628

[B54] Lobito A. A. Kimberley F. C. Muppidi J. R. Komarow H. Jackson A. J. Hull K. M.. (2006). Abnormal disulfide-linked oligomerization results in ER retention and altered signaling by TNFR1 mutants in TNFR1-associated periodic fever syndrome (TRAPS). Blood 108, 1320–1327. 10.1182/blood-2005-11-006783 16684962 PMC1895878

[B55] Magrané J. Hervias I. Henning M. S. Damiano M. Kawamata H. Manfredi G. (2009). Mutant SOD1 in neuronal mitochondria causes toxicity and mitochondrial dynamics abnormalities. Hum. Mol. Genet. 18, 4552–4564. 10.1093/hmg/ddp421 19779023 PMC2773270

[B56] McAlary L. Yerbury J. J. Aquilina J. A. (2013). Glutathionylation potentiates benign superoxide dismutase 1 variants to the toxic forms associated with amyotrophic lateral sclerosis. Sci. Rep. 3:3275. 10.1038/srep03275 24253732 PMC3834562

[B202] Medraño-Fernandez I. Bestetti S. Bertolotti M. Bienert G. P. Bottino C. Laforenza U.. (2016). Stress regulates aquaporin-8 permeability to impact cell growth and survival. Antioxid. Redox Signal. 24, 1031–1044. 10.1089/ars.2016.6636 26972385 PMC4931348

[B57] Medraño-Fernandez I. Fagioli C. Mezghrani A. Otsu M. Sitia R. (2014). Different redox sensitivity of endoplasmic reticulum associated degradation clients suggests a novel role for disulphide bonds in secretory proteins. Biochem. Cell Biol. 92, 113–118. 10.1139/bcb-2013-0090 24697695

[B58] Meng F. Yao D. Shi Y. Kabakoff J. Wu W. Reicher J.. (2011). Oxidation of the cysteine-rich regions of parkin perturbs its E3 ligase activity and contributes to protein aggregation. Mol. Neurodegener. 6:34. 10.1186/1750-1326-6-34 21595948 PMC3120712

[B59] Mesecke N. Terziyska N. Kozany C. Baumann F. Neupert W. Hell K.. (2005). A disulfide relay system in the intermembrane space of mitochondria that mediates protein import. Cell 121, 1059–1069. 10.1016/j.cell.2005.04.011 15989955

[B60] Mishanina T. V. Libiad M. Banerjee R. (2015). Biogenesis of reactive sulfur species for signaling by hydrogen sulfide oxidation pathways. Nat. Chem. Biol. 11, 457–464. 10.1038/nchembio.1834 26083070 PMC4818113

[B61] Mishra M. Jiang H. Wu L. Chawsheen H. A. Wei Q. (2015). The sulfiredoxin-peroxiredoxin (Srx-Prx) axis in cell signal transduction and cancer development. Cancer Lett. 366, 150–159. 10.1016/j.canlet.2015.07.002 26170166 PMC4532351

[B62] Molteni S. N. Fassio A. Ciriolo M. R. Filomeni G. Pasqualetto E. Fagioli C.. (2004). Glutathione limits Ero1-dependent oxidation in the endoplasmic reticulum. J. Biol. Chem. 279, 32667–32673. 10.1074/jbc.M404992200 15161913

[B63] Mulligan L. M. (2014). RET revisited: expanding the oncogenic portfolio. Nat. Rev. Cancer 14, 173–186. 10.1038/nrc3680 24561444

[B64] Mustacich D. Powis G. (2000). Thioredoxin reductase. Biochem. J. 346, 1–8. 10.1042/0264-6021:3460001 10657232 PMC1220815

[B65] Nagy P. (2013). Kinetics and mechanisms of thiol-disulfide exchange covering direct substitution and thiol oxidation-mediated pathways. Antioxid. Redox Signal. 18, 1623–1641. 10.1089/ars.2012.4973 23075118 PMC3613173

[B66] Nakamura T. Lipton S. A. (2013). Emerging role of protein-protein transnitrosylation in cell signaling pathways. Antioxid. Redox Signal. 18, 239–249. 10.1089/ars.2012.4703 22657837 PMC3518546

[B67] Nakamura T. Lipton S. A. (2016). Protein S-Nitrosylation as a therapeutic target for neurodegenerative diseases. Trends Pharmacol. Sci. 37, 73–84. 10.1016/j.tips.2015.10.002 26707925 PMC4698225

[B68] Numajiri N. Takasawa K. Nishiya T. Tanaka H. Ohno K. Hayakawa W.. (2011). On-off system for PI3-kinase-Akt signaling through S-nitrosylation of phosphatase with sequence homology to tensin (PTEN). Proc. Natl. Acad. Sci. U S A 108, 10349–10354. 10.1073/pnas.1103503108 21646525 PMC3121815

[B69] Okumura M. Kadokura H. Inaba K. (2015). Structures and functions of protein disulfide isomerase family members involved in proteostasis in the endoplasmic reticulum. Free Radic. Biol. Med. 83, 314–322. 10.1016/j.freeradbiomed.2015.02.010 25697777

[B70] Oteiza P. I. (2012). Zinc and the modulation of redox homeostasis. Free Radic. Biol. Med. 53, 1748–1759. 10.1016/j.freeradbiomed.2012.08.568 22960578 PMC3506432

[B71] Patel K. A. Bartoli K. M. Fandino R. A. Ngatchou A. N. Woch G. Carey J.. (2005). Transmembrane S1 mutations in CNGA3 from achromatopsia 2 patients cause loss of function and impaired cellular trafficking of the cone CNG channel. Invest. Ophthalmol. Vis. Sci. 46, 2282–2290. 10.1167/iovs.05-0179 15980212

[B72] Paulsen C. E. Carroll K. S. (2013). Cysteine-mediated redox signaling: chemistry, biology and tools for discovery. Chem. Rev. 113, 4633–4679. 10.1021/cr300163e 23514336 PMC4303468

[B73] Perkins A. Nelson K. J. Parsonage D. Poole L. B. Karplus P. A. (2015). Peroxiredoxins: guardians against oxidative stress and modulators of peroxide signaling. Trends Biochem. Sci. 40, 435–445. 10.1016/j.tibs.2015.05.001 26067716 PMC4509974

[B74] Poet G. J. Oka O. B. van Lith M. Cao Z. Robinson P. J. Pringle M. A.. (2017). Cytosolic thioredoxin reductase 1 is required for correct disulfide formation in the ER. EMBO J. 36, 693–702. 10.15252/embj.201695336 28093500 PMC5331760

[B75] Pulido R. (2015). PTEN: a yin-yang master regulator protein in health and disease. Methods 77–78, 3–10. 10.1016/j.ymeth.2015.02.009 25843297

[B76] Rampoldi L. Caridi G. Santon D. Boaretto F. Bernascone I. Lamorte G.. (2003). Allelism of MCKD, FJHN and GCKD caused by impairment of uromodulin export dynamics. Hum. Mol. Genet. 12, 3369–3384. 10.1093/hmg/ddg353 14570709

[B77] Redler R. L. Wilcox K. C. Proctor E. A. Fee L. Caplow M. Dokholyan N. V. (2011). Glutathionylation at Cys-111 induces dissociation of wild type and FALS mutant SOD1 dimers. Biochemistry 50, 7057–7066. 10.1021/bi200614y 21739997 PMC3281512

[B78] Ronzoni R. Berardelli R. Medicina D. Sitia R. Gooptu B. Fra A. M. (2016). Aberrant disulphide bonding contributes to the ER retention of alpha1-antitrypsin deficiency variants. Hum. Mol. Genet. 25, 642–650. 10.1093/hmg/ddv501 26647313

[B79] Rouault T. A. (2015). Mammalian iron-sulphur proteins: novel insights into biogenesis and function. Nat. Rev. Mol. Cell Biol. 16, 45–55. 10.1038/nrm3909 25425402

[B80] Scolari F. Izzi C. Ghiggeri G. M. (2015). Uromodulin: from monogenic to multifactorial diseases. Nephrol. Dial. Transplant. 30, 1250–1256. 10.1093/ndt/gfu300 25228753

[B81] Seirafi M. Kozlov G. Gehring K. (2015). Parkin structure and function. FEBS J. 282, 2076–2088. 10.1111/febs.13249 25712550 PMC4672691

[B82] Sen N. Snyder S. H. (2011). Neurotrophin-mediated degradation of histone methyltransferase by *S*-nitrosylation cascade regulates neuronal differentiation. Proc. Natl. Acad. Sci. U S A 108, 20178–20183. 10.1073/pnas.1117820108 22123949 PMC3250167

[B83] Siegenthaler K. D. Pareja K. A. Wang J. Sevier C. S. (2017). An unexpected role for the yeast nucleotide exchange factor Sil1 as a reductant acting on the molecular chaperone BiP. Elife 6:e24141. 10.7554/eLife.24141 28257000 PMC5358974

[B84] Smith J. K. Patil C. N. Patlolla S. Gunter B. W. Booz G. W. Duhé R. J. (2012). Identification of a redox-sensitive switch within the JAK2 catalytic domain. Free Radic. Biol. Med. 52, 1101–1110. 10.1016/j.freeradbiomed.2011.12.025 22281400 PMC3319112

[B85] Takahashi M. Iwashita T. Santoro M. Lyonnet S. Lenoir G. M. Billaud M. (1999). Co-segregation of MEN2 and Hirschsprung’s disease: the same mutation of RET with both gain and loss-of-function? Hum. Mutat. 13, 331–336. 10.1002/(SICI)1098-1004(1999)13:4331::AID-HUMU11>3.0.CO;2-# 10220148

[B86] Uehara T. Nakamura T. Yao D. Shi Z.-Q. Gu Z. Ma Y.. (2006). *S*-nitrosylated protein-disulphide isomerase links protein misfolding to neurodegeneration. Nature 441, 513–517. 10.1038/nature04782 16724068

[B87] Ushioda R. Hoseki J. Araki K. Jansen G. Thomas D. Y. Nagata K. (2008). ERdj5 is required as a disulfide reductase for degradation of misfolded proteins in the ER. Science 321, 569–572. 10.1126/science.1159293 18653895

[B88] Valle C. Carrì M. T. (2017). Cysteine modifications in the pathogenesis of ALS. Front. Mol. Neurosci. 10:5. 10.3389/fnmol.2017.00005 28167899 PMC5253364

[B200] Vavassori S. Cortini M. Masui S. Sannino S. Anelli T. Caserta I. R.. (2013). A pH-regulated quality control cycle for surveillance of secretory protein assembly. Mol. Cell 50, 783–792. 10.1016/j.molcel.2013.04.016 23685074 PMC3699783

[B89] Venereau E. Casalgrandi M. Schiraldi M. Antoine D. J. Cattaneo A. De Marchis F.. (2012). Mutually exclusive redox forms of HMGB1 promote cell recruitment or proinflammatory cytokine release. J. Exp. Med. 209, 1519–1528. 10.1084/jem.20120189 22869893 PMC3428943

[B90] Vénéreau E. Ceriotti C. Bianchi M. E. (2015). DAMPs from cell death to new life. Front. Immunol. 6:422. 10.3389/fimmu.2015.00422 26347745 PMC4539554

[B91] Visscher M. Arkin M. R. Dansen T. B. (2016). Covalent targeting of acquired cysteines in cancer. Curr. Opin. Chem. Biol. 30, 61–67. 10.1016/j.cbpa.2015.11.004 26629855 PMC4731306

[B92] Wagener K. C. Kolbrink B. Dietrich K. Kizina K. M. Terwitte L. S. Kempkes B.. (2016). Redox indicator mice stably expressing genetically encoded neuronal roGFP: versatile tools to decipher subcellular redox dynamics in neuropathophysiology. Antioxid. Redox Signal. 25, 41–58. 10.1089/ars.2015.6587 27059697 PMC4931743

[B95] Wang J.-W. Groeneveld D. J. Cosemans G. Dirven R. J. Valentijn K. M. Voorberg J. (2012). Biogenesis of Weibel-Palade bodies in von Willebrand’s disease variants with impaired von Willebrand factor intrachain or interchain disulfide bond formation. Haematologica 97, 859–866. 10.3324/haematol.2011.057216 22207689 PMC3366651

[B94] Wang J. Pareja K. A. Kaiser C. A. Sevier C. S. (2014). Redox signaling via the molecular chaperone BiP protects cells against endoplasmic reticulum-derived oxidative stress. Elife 3:e03496. 10.7554/eLife.03496 25053742 PMC4132286

[B93] Wang C. Tan J. M. M. Ho M. W. L. Zaiden N. Wong S. H. Chew C. L. C.. (2005). Alterations in the solubility and intracellular localization of parkin by several familial Parkinson’s disease-linked point mutations. J. Neurochem. 93, 422–431. 10.1111/j.1471-4159.2005.03023.x 15816865

[B96] Wani R. Murray B. W. (2017). Analysis of cysteine redox post-translational modifications in cell biology and drug pharmacology. Methods Mol. Biol. 1558, 191–212. 10.1007/978-1-4939-6783-4_9 28150239

[B97] Wei P.-C. Hsieh Y.-H. Su M.-I. Jiang X. Hsu P.-H. Lo W.-T.. (2012). Loss of the oxidative stress sensor NPGPx compromises GRP78 chaperone activity and induces systemic disease. Mol. Cell 48, 747–759. 10.1016/j.molcel.2012.10.007 23123197 PMC3582359

[B98] Woo H. A. Yim S. H. Shin D. H. Kang D. Yu D.-Y. Rhee S. G. (2010). Inactivation of peroxiredoxin I by phosphorylation allows localized H_2_O_2_ accumulation for cell signaling. Cell 140, 517–528. 10.1016/j.cell.2010.01.009 20178744

[B99] Zhang Z. Liu L. Jiang X. Zhai S. Xing D. (2016). The essential role of Drp1 and its regulation by S-nitrosylation of parkin in dopaminergic neurodegeneration: implications for Parkinson’s disease. Antioxid. Redox Signal. 25, 609–622. 10.1089/ars.2016.6634 27267045

[B100] Zhao Q.-F. Yu J.-T. Tan L. (2015). *S*-Nitrosylation in Alzheimer’s disease. Mol. Neurobiol. 51, 268–280. 10.1007/s12035-014-8672-2 24664522

[B101] Zheng M. Aslund F. Storz G. (1998). Activation of the OxyR transcription factor by reversible disulfide bond formation. Science 279, 1718–1721. 10.1126/science.279.5357.1718 9497290

